# Detection of Porcine Circovirus Type 2a and *Pasteurella multocida* Capsular Serotype D in Growing Pigs Suffering from Respiratory Disease

**DOI:** 10.3390/vetsci9100528

**Published:** 2022-09-27

**Authors:** Shuailong Du, Fan Xu, Yidan Lin, Yawen Wang, Yanan Zhang, Kai Su, Tanqing Li, Huanrong Li, Qinye Song

**Affiliations:** 1 Hebei Veterinary Biotechnology Innovation Center, College of Veterinary Medicine, Hebei Agricultural University, Baoding 071000, China; 2 College of Animal Science and Technology, Beijing University of Agriculture, Beijing 102206, China

**Keywords:** respiratory disease, porcine circovirus type 2a, *P. multocida* capsular serotypes D, co-infection, growing pigs

## Abstract

**Simple Summary:**

Bacterial and viral respiratory diseases are a major health problem in pig farms. Most often multiple infectious agents are involved in the development of clinical cases of respiratory diseases. It is necessary to conduct laboratory etiological tests on suspected cases. In January 2021, a 70-day-old pig herd in a pig farm in Hebei Province, China, had been suffering from respiratory problems. On the basis of this preliminary clinical diagnosis, this study carried out comprehensive laboratory detection including nucleic acid detection and sequencing, the isolation and identification of the pathogen, genetic evolution analysis, and genotype or serotype identification, as well as an analysis of the bacterial morphology characteristics and pathogenicity. The results showed that the co-infection of PCV2a and *P. multocida* capsular serotypes D was likely responsible for the disease. This study could provide important etiological information for effective control and treatment of the disease in pig farms.

**Abstract:**

In order to diagnose a respiratory disease in a pig farm, the lungs, spleen, and lymph nodes of three dead pigs were collected for pathogen detection by PCR and isolation on the basis of preliminary clinical diagnosis. The virus isolate was identified by gene sequence analysis and Immunoperoxidase monolayer assay (IPMA). The bacterial isolate was identified by biochemical tests, 16S rDNA sequence analysis, and species- and serotype-specific PCR, and the pathogenicity was analyzed. Porcine circovirus type 2a (PCV2a) genotype from the lungs, spleen, and lymph nodes and *Pasteurella (P.)* *multocida* capsular serotypes D from the lungs were found. The PCV2a isolates could specifically bound the anti-PCV2-Cap polyclonal antibody. The 16S rDNA sequence of *P. multocida* isolates had 99.9% identity with that of the strain from cattle, and the isolate was highly pathogenic to mice. The results showed that the co-infection of PCV2a and *P. Multocida* capsular serotypes D should be responsible for the disease. The uncommon PCV2a is still prevalent in some pig farms besides the dominant PCV2d genotype. This study could provide important etiological information for effective control and treatment of the disease in pig farms.

## 1. Introduction

Porcine circovirus type 2 (PCV2), a naked circular single-stranded DNA virus with a 1767~1768 bp genome, belongs to genus *Circovirus* in the family *Circoviridae* [1]. The virus is responsible for porcine circovirus disease (PCVD) or porcine circovirus-associated disease (PCVAD) which is an immune suppression disease characterized as a clinical or subclinical infection [2]. PCVAD includes a variety of multi-symptomatic diseases such as porcine circovirus 2-systemic disease (PCV2-SD), formerly known as postweaning multisystemic wasting syndrome (PMWS), porcine dermatitis and nephropathy syndrome (PDNS), porcine respiratory disease syndrome (PRDC), and reproductive disorders, of which PCV2-SD is the most common disease in clinical practice [3,4]. Co-infection with other pathogens can promote PCV2 replication in the host, resulting in subclinical infection of PCV2 to clinical infection (clinical PCVD) [5,6]. The genome of PCV2 has a high mutation [7] and eight genotypes (PCV2a-2h) have been identified since the virus was first identified in 1998 [8,9,10]. However, the dominant genotype of PCV2 prevalent in pig farms has been shifting in different periods, such as PCV2a from 1996 to the early 2000s, then PCV2b from about 2005, and PCV2d in most pig farms from around 2012 [8,11]. The change in PCV2 genotype may favor survival, antigenicity, pathogenicity, and circulation of the virus, which poses a new challenge to vaccination for the prevention of PCVD [11]. At present, PCV2 is widely distributed in pig farming areas all over the world, which causes strong direct and indirect economic impact on the pig industry every year.

Respiratory diseases, PRDC, caused by more than one type of pathogen (viruses, bacteria, parasites, and fungi) is a major and serious health issue on pig farms along with the large-scale and intensive development of the pig industry. Combinations of several infectious pathogens, in particular viruses and bacteria, frequently occur and lead to PRDC in current pig farms [12,13]. Porcine reproductive and respiratory syndrome virus (PRRSV), porcine circovirus type 2 (PCV2), pseudorabies virus (PRV), swine influenza A virus (swIAV), and *Mycoplasma (M.) hyopneumoniae*, *Actinobacillus (A.) pleuropneumoniae*, *Pasteurella (P.) multocida*, *Streptococcus (S.) suis*, or *Bordetella (B.) bronchiseptica* are frequently detected in cases of PRDC [12,14,15,16,17]. However, the type of combinations and kind of associated infectious agents vary with local weather conditions, region, and breeding conditions, which generally complicates the severity, prevention, and control of the disease [18]. *P. multocida* is one of the most common bacterial agents isolated from respiratory clinical cases worldwide and often is a secondary or opportunistic pathogen of PRDC [19,20]. Five capsular serotypes (A, B, D, E and F) are recognized, with A and D comprising most swine isolates [21,22]. However, the prevalent *P. multocida* serotypes can vary considerably from region to region and over time in a given region. Therefore, laboratory testing is often required to determine the causes and make an accurate diagnosis in order to provide guidance for disease control.

In January 2021, a 70-day-old pig herd in a pig farm in Hebei Province, China, had been suffering from respiratory problems. In order to diagnose and help to control the disease, this study carried out comprehensive laboratory detection on the basis of the preliminary clinical diagnosis, including nucleic acid detection and sequencing, isolation and identification of pathogen, genetic evolution analysis, and genotype or serotype identification, as well as an analysis bacterial morphology characteristics and pathogenicity.

## 2. Materials and Methods

### 2.1. Case Presentation and Sample Collection

Some of 70-day-old growing pigs in a pig farm had been suffering from respiratory disease with clinical manifestations including a cough, abdominal breathing or dyspnea, intermittent fever (40–41 °C), depression, anorexia, and in severe cases cyanosis in the ear, neck, and abdomen. About 20% of the pigs in the same herd showed similar clinical manifestations. It took more than ten days for most pigs to go through the onset of clinical signs to death, but a few sick pigs died quickly. Five had died of the disease in recent days. In the necropsy of dead pigs, obvious gross lesions of red-to-gray consolidation in the lungs, and a large number of foamy secretions in the trachea, bronchus, and alveolar cavity were observed. In some cases, pericarditis and liver and spleen congestion were seen. The herd had been inoculated with attenuated classical swine fever virus (CSFV) and PRV vaccines, but not with any type of PCV2 and PRRSV vaccine. On the basis of clinical diagnosis, lung, spleen, and lymph-node samples were collected for laboratory testing.

### 2.2. Viral Detection by PCR

An appropriate amount of lung, lymph-node, and spleen tissues were added to PBS at a weight/volume ratio of 1:3 to make tissue homogenate. Centrifuged at 8000 r/min for 10 min, the supernatant was collected and total viral nucleic acids were extracted from tissues using a Viral Total Nucleic Acid Purification kit (Tiangen Biotech, Beijing, China) according to the manufacturer’s instructions. PCV2, PCV3, PRRSV, and PRV detection were performed by PCR or RT-PCR with specific primers (Table 1) in an Applied Biosystem Veriti 9902 PCR System. The PCR was run according to the following protocol: 2 × Taq Master Mix 10 μL (CWBIO, Beijing, China), 25 μM of each primer 0.5 μL, DNA or cDNA 2 μL and ddH_2_O 7 μL. The amplification procedure was as follows: pre-denaturing for 4 min at 94 °C, followed by 35 cycles of denaturation for 20 s at 94 °C, annealing at 56–57 °C (PCV2, PCV3 or PRRSV) or 67 °C (PRV) for 30 s, extension at 72 °C for 45 s, and a final extension at 72 °C for 5 min. The amplified products were detected by 1.5% agarose gel electrophoresis.

### 2.3. Virus Isolation and Identification

The spleen, lymph nodes, and lungs were cut into pieces and homogenized to make tissue suspension after adding sterile PBS according to a ratio of tissue weight (g) to volume (V) of 1:3. After the tissue homogenate was freeze-thawed three times, centrifugation was performed at 8000 r/min for 10 min. The supernatant was collected, penicillin and streptomycin 1000 U/mL were each added, and then it was placed at 4 °C overnight, filtered by a 0.22 μm filter for sterilization, and then used for virus isolation and culture.

At the same time of the passage of PCV1-free PK-15 cells, 1 mL of the treated tissue supernatant was inoculated and cultured at 37 °C with 5% CO2 for 48–72 h. The virus isolates designated HBSZ-2021 was collected and continued through passages in PK-15 cells. PCR was employed to detect the isolate with primers PCV2_cap_U-PCV2_cap_L. At the same time, the immunoperoxidase monolayer assay (IPMA) was also used to identify the virus isolate [25]. At the same time, negative serum and blank cell controls were set up. The anti-PCV2 specific positive and negative serum used in IPMA were mouse anti-PCV2-Cap polyclonal antibody and non-immunized mouse serum (1:200 diluted) prepared in our laboratory, and HRP-labeled sheep anti-mouse IgG (1:300 diluted) (Beijing CW Biotech Co., Ltd., Beijing, China).

### 2.4. Viral Genome Amplification and Phylogenetic Analysis

Total viral DNA was extracted from the above tissue homogenates and the viral isolate using Virus Genomic DNA Isolation Kit (Tiangen Biotech, Beijing, China) according to the manufacturer’s protocol. The complete PCV2 genome sequence was amplified by PCR with primers (U-PCV2 and L-PCV2) (Table 1). PCR was performed according to the thermal cycling conditions in reference. PCR products were recovered with Agarose Gel DNA Recovery Kit (Tiangen Biotech, Beijing, China) and ligated into the pMD18-T vector (TaKaRa) using T4 DNA ligase (Promega). The target gene which was cloned into the plasmids was sequenced by Sangon Biotech (Shanghai) Co., Ltd., Shanghai, China.

The obtained PCV2 strain (HBSZ-2021) genome sequence (GenBank ID: MZ161162) and 23 reference sequences belonged to different genotype of PCV2 in GenBank (Table 2) were aligned by the MegAlign program of the DNASTAR package version 7.10 with the Clustal W method (DNASTAR, Madison, WI, USA). The identities of the genome, ORF1, ORF2, and the deduced amino acid sequence of ORF2 were analyzed among the virus with the reference strains. Phylogenetic trees based on the entire genomes and nucleotide and deduced amino acid (aa) sequences of ORF2 were constructed using the neighbor-joining method of MEGA6.06 with a bootstrap of 1000 replicate datasets. The viral genotype was subsequently determined according to the phylogenetic trees.

### 2.5. Bacterial Isolation and Culture

Before bacterial isolation and culture, a bacterial genomic DNA extraction kit (Tiangen Biotech, Beijing, China) was used to extract bacterial genomic DNA from lung tissue. *M. hyopneumoniae* was excluded by PCR using specific primers in Table 1. Following this, the lung, spleen, and liver samples were taken and streaked onto ordinary nutrient agar (Beijing Biotech AOB Star Co., Beijing, China) and serum–agar plates with 5% fetal bovine serum (FBS, Gibco) in ordinary nutrient agar medium. Colony morphology was examined, Gram or Wright stained, and the morphology of bacteria staining was observed under a microscope after 18~24 h incubation at 37 °C. The single colony was re-streaked onto the ordinary nutrient agar, serum–agar, nutrient agar containing 5% fresh rabbit blood, and MacConkey agar (AOBOX, Beijing, China), and incubated 24 h at 37 °C. The bacterial culture and phenotypic characteristics of colonies were further observed. The morphological characteristics of bacteria after Gram or Wright staining were examined under a microscope.

### 2.6. Bacterial Catalase and Biochemical Tests

To determine whether the isolated bacteria produced catalase, the catalase test was carried out according in reference [26]. The isolated bacterial colonies were picked and placed on clean glass slides. At the same time, the strain ATCC25923 of *Staphylococcus aureus* and the strain ATCC 27335 of *Streptococcus intermedius* was used as the positive or the negative control, respectively. Furthermore, 3% hydrogen peroxide (Sigma) was dropped onto the bacteria on the slides. When large bubbles occurred, the catalase was positive; otherwise it was negative.

Meanwhile, the biochemical test was conducted. Briefly, the isolated bacteria were inoculated into biochemical identification microtubes (Hangzhou Binhe Microorganism Regent Co., Ltd., Hangzhou, China) and cultured for 48 h at 37 °C according to the manufacturer’s instructions. The biochemical reaction results were checked and analyzed according to Bergey’s Manual of Systematic Bacteriology (9th edition).

### 2.7. Identification of Bacterial Species Based on 16S rDNA Sequences

To identify the species of isolated bacteria, the 16S rRNA gene (rDNA) sequence from the purified bacterial isolates were amplified by PCR with universal primers 16SF (5′-AGAGTTTGATCCTGGCTCAG-3′) and 16SR (5′-GGTTACCTTGTTACGACTT-3′) [27]. The PCR products were sequenced at Sangon Biotech (Shanghai) Co., Ltd., China. The 16S rDNA sequence from the isolate, designated as HBShz_2021 (GenBank ID: OK236057), was compared with the reference sequences available in GenBank (Table 3) and an unrooted phylogenetic tree was constructed using the neighbor-joining method of MEGA6.06.

### 2.8. PCR Identification of Bacterial Species and Capsular Serotypes

The species and serotype of the bacterial isolate were identified by PCR using the species- and capsular-type (A, B, D, E and F) specific primes of *P. multocida*, respectively [28] (Table 4). A 1.5 mL quantity of bacterial cultures was taken to centrifuge at 5000 r/min for 5 min. The bacterial cells were collected, resuspended with 500 μL sterile water and mixed thoroughly. The mixture was boiled at 100 °C for 10 min to release DNA and centrifuged at 12,000 r/min for 10 min. The supernatant was collected as DNA templates for PCR. A 2 μL quantity of the supernatant was used as a template for each 20 µL PCR mixture containing 10 μL of 2 × Taq Master Mix, 0.5 μL of each primer (25 μM), and 7 μL ddH_2_O. The following cycling procedure was used: an initial denaturation at 94 °C for 4 min, followed by 35 cycles of denaturation at 94 °C for 45 s, annealing at 55 °C for 45 s, extension at 72 °C for 45 s, and a final extension at 72 °C for 10 min.

### 2.9. Pathogenicity Test of the Isolated Bacteria

The isolated bacteria were inoculated into nutrient broth containing 5% FBS at 37 °C for 24 h and subsequently colony-forming units (CFU) were counted. An animal experiment was referenced [29], but with modifications. Six 4-week-old SPF Kunming mice were each injected intraperitoneally with 2 × 10^8^ CFU of the bacteria. Three mice were set up as negative control, and each was injected intraperitoneally with an equal volume of nutritional broth containing 5% FBS. After infection, the clinical manifestations were observed every day, the morbidity and mortality were recorded, and the gross lesions of the dead mice were examined. Microscopic examination of the bacteria was implemented in lung and live tissues after Gram and Wright staining. At the same time, the tissues were streaked onto nutrient agar containing 5% FBS, and the morphology of colony or bacteria after 24 h culture at 37 °C.

## 3. Results

### 3.1. PCV2 Nucleic Acid Was Detected in the Tissue

**Figure 1:**
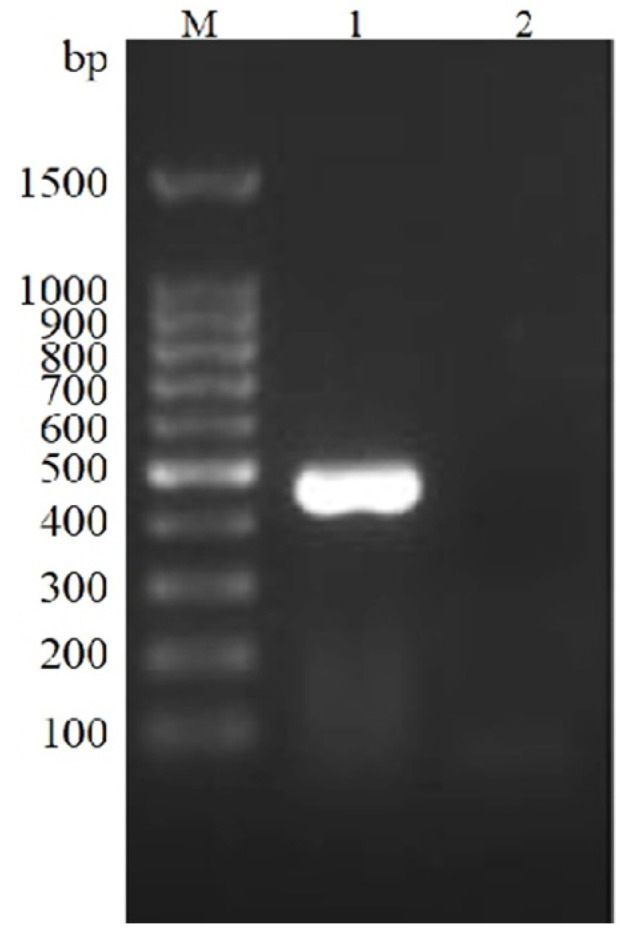
Detection of PCV2 ORF2 by PCR. M: DNA Ladder; 1: Lungs; 2: Negative control.

The 472 bp specific nucleotide sequences of PCV2 ORF2 were amplified by PCR from the lung, lymph-node, and spleen tissues of diseased pigs (Figure 1). No expected nucleotide of PCV3-ORF2, PRRSV-ORF7, or PRV-gE genes was amplified. The results showed that PCV2 was associated with the disease, but not with PCV3, PRRSV, and PRV.

### 3.2. The PCV2 Strain Was Isolated from the Tissue

**Figure 2:**
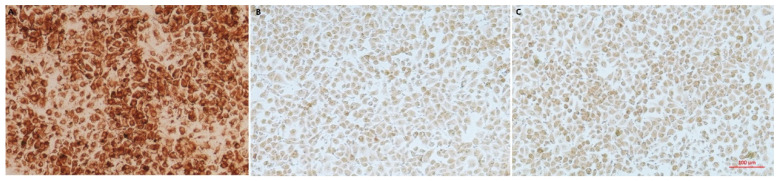
Identification of the virus isolate with immunoperoxidase monolayer assay (IPMA). (**A**) The isolate; (**B**) Negative serum against PCV2; (**C**) Blank control.

No cytopathic effects (CPE) were observed 72–96 h after the inoculation of the treated spleen and lymph-node homogenates into PK15 cells and following continuous transmission of the isolate for seven passages. The PCV2 nucleotide sequence corresponding to the expected size (472 bp) was amplified from the isolated culture by PCR. Specific brown response signals were observed in PK15 cells inoculated with the seventh isolate passage (mainly in the cytoplasm) by IPMA detection, while there were no similar signals in the negative control and blank control cells (Figure 2), indicating PCV2 (named HBSZ-2021 strain) proliferation occurred in PK15 cells inoculated with this isolate.

### 3.3. Sequence Analysis and Phylogenetic Tree of the PCV2 Isolate

The same 1768 bp PCV2 genomic sequences (GenBank ID: MZ161162) were amplified by PCR from the spleen and lymph node tissue samples. The genomic sequence of the isolate contained two main open reading frames ORF1 and ORF2, which consisted of 945 bp encoding 314 aa and 702 bp encoding 233 aa, respectively. The identity of complete genome sequences between HBSZ-2021 strain and the reference PCV2 strains was 93.5~97.5%, in which the identity with genotypes PCV2a, PCV2b, PCV2c, and PCV2d was 96.5~97.5%, 94.8~95.8%, 93.5~93.6%, and 93.5~94.9%, respectively (Supplementary Figure S1).

The homology of the PCV2 HBSZ-2021 strain ORF1with PCV2a, PCV2b, PCV2c, and PCV2d was 98–98.7%, 97.1–98.2%, 98.3%, and 96.9–97.5%, respectively (Supplementary Figure S2). The homology of the amino acid sequence it encoded was 99.4–100%, 98.7–100%, 98.7–99%, and 98.4–99.4% with those encoded by PCV2a, PCV2b, PCV2c, and PCV2d reference strains, respectively (Supplementary File S2). Although there were base substitutions in ORF1 among PCV2 strains of different genotypes, most of them were same sense mutations. This indicated that ORF1 had high similarity among various PCV2 strains, among which the HBSZ-2021 strain had the highest similarity with the PCV2a strain.

In contrast, ORF2 showed low similarity among different PCV2 genotypes, with multiple single- and continuous multi-base mutations, as well as base-elongation mutations. The identities of ORF2 between HBSZ-2021 strain and PCV2a, PCV2b, PCV2c, and PCV2d reference strains were as follows: 94.3% to 95.3%, 90.7% to 92.2%, 85.8% to 86.0%, and 89.2% to 90.6%. The similarities of the amino acid sequence encoded by it with those encoded by PCV2a, PCV2b, PCV2c, and PCV2d reference strains were 91.0–95.3%, 88.4–90.1%, 82.8–83.3%, and 87.1–88.8%, respectively (Supplementary Figure S3). The above data showed that the HBSZ-2021 strain had the highest identities with PCV2a ORF1 and ORF2 nucleotide sequences and the amino acid sequences encoded by them.

**Figure 3:**
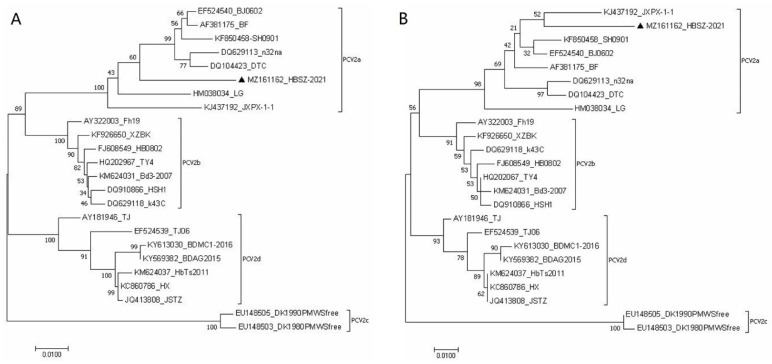
Phylogenetic trees of PCV2 HBSZ-2021 strain and the reference strains based on the ORF2 nucleotide and amino acid sequences. (**A**) Nucleotide sequences; (**B**) Amino acid sequences. The information of PCV2 reference strains is shown in Table 2. ▲ The detected strain HBSZ-2021.

The phylogenetic trees displayed that the PCV2 HBSZ-2021 strain clustered in the same large branch with the PCV2a strains originating from different regions based on the viral whole genome sequence and the nucleotide and amino acid sequences of ORF2, indicating that the HBSZ-2021 strain was more closely related to the PCV2a than to the other genotype strains such as PCV2b, PCV2c, and PCV2d strains. The results showed that the HBSZ-2021 strain belonged to the PCV2a genotype (Supplementary Figure S4 and Figure 3).

### 3.4. Characteristics of Bacterial Culture and Morphology

**Figure 4:**
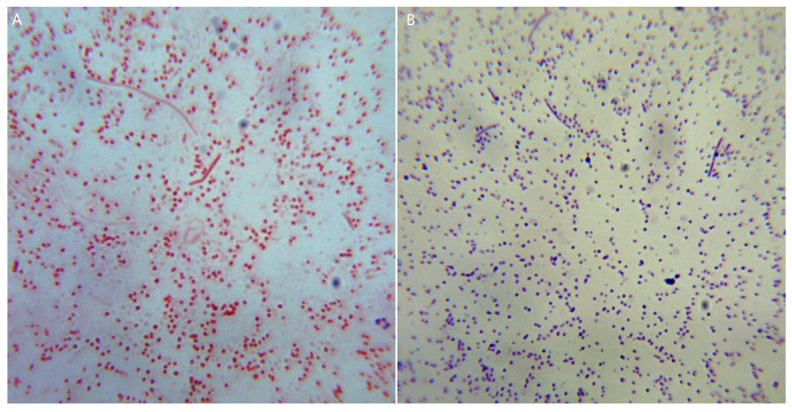
Morphological characteristics of isolated bacteria under microscope (×1000). (**A**) Gram stain; (**B**) Wright stain.

In order to determine whether the infection involved bacteria, bacterial isolation, culture, and a morphological examination were carried out on the tissue samples. Bacteria were isolated from the lungs of sick pigs and grew well on the nutrient agar containing 5% calf serum or 5% rabbit blood, but poorly on the nutrient agar and did not grow on McConkey agar. After streaking inoculation on serum or blood agar medium for 24 h, moist, grayish-white dewdrop-like colonies with a round, smooth surface were grown. There was no hemolysis on the blood agar medium (Supplementary Figure S5). Microscopy analysis displayed that the isolated bacteria were obtuse at both ends, showing a typical two-polarity dense-stained Gram-negative short bacillus, and mostly scattered in distribution, occasionally in a short filamentous arrangement (Figure 4).

### 3.5. Biochemical Characteristics of Bacteria

To determine the biochemical characteristics of the isolated bacteria, the study performed biochemical experiments. After dropping 3% hydrogen peroxide solution onto the isolated bacteria, many bubbles emerged, which indicated that the isolated strains were catalase-positive. Further biochemical assays showed that the isolated bacteria could ferment sucrose, mannitol, xylose, and sorbitol, but not glucose, lactose, maltose, dulcitol, adonitol, or raffinose. They could produce hydrogen sulfide and indole, but not urease, phenylalanine, ornithine, or lysine decarboxylase. The isolate displayed negative responses to methyl red (MR) and Voges–Proskaurer (V-P) tests, as well as citrate utilization test (Table 5). These results are consistent with the main biochemical characteristics of *P. multocida*.

### 3.6. Phylogenetic Analysis of the Isolated Bacteria Based on 16S rDNA Sequences

**Figure 5:**
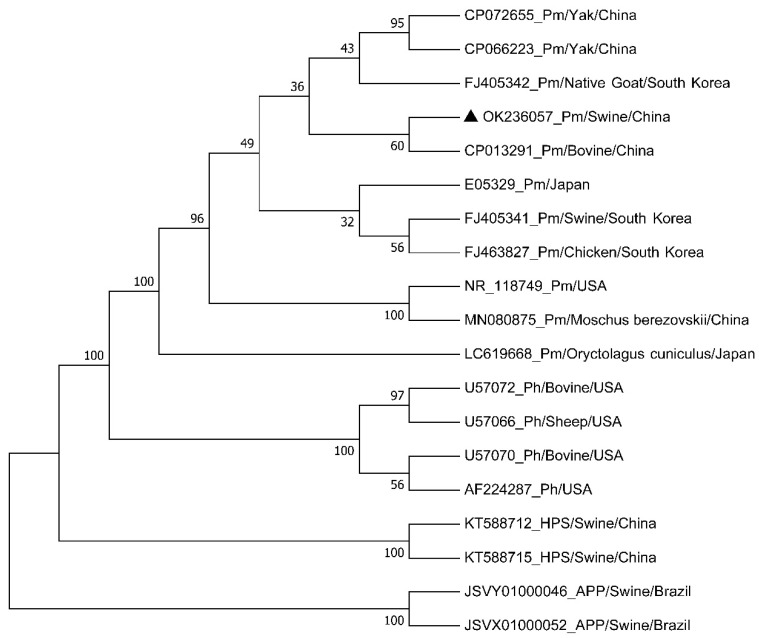
Phylogenetic trees of the bacterial isolate and the reference strains based on 16S rDNA sequences. The information of the reference strains is shown in Table 3. Pm: *P. multocida*; Ph: *P. hemolyticus*; HPS: *H. parasuis*; APP: *A. pleuropneumoniae;* ▲ The bacterial isolate HBShz_2021.

The 16S rDNA sequence was amplified by PCR from the genome of isolated bacteria for the phylogenetic analysis. Sequencing and sequence analysis showed that the full length of the amplicons was 1593 bp (named HBShz_2021, GenBank ID: OK236057), which had a 92.5~99.9% identity with the 16S rDNA sequences of *Pasteurella*, 95.3–99.9% with *P. multocida*, and 92.5–94.5% with *P. hemolyticus* (Supplementary Figure S6). However, the consistency of the isolates with *H. parasuis* and *A. pleuropneumoniae* strains was 38.2~39.9% and 35.8%, respectively. A phylogenetic tree based on bacterial 16S rDNA sequences displayed that *Pasteurella*, *H. parasuis*, and *A. pleuropneumoniae* were in different genetic evolutionary branches of the phylogenetic tree, in which *Pasteurella* was divided into two branches: *P. multocida* and *P. hemolyticus* (Figure 5). The isolated strain was clustered in the same evolutionary clade with *P. multocida* strains originated from pigs, cattle, sheep, and chickens. Moreover, the isolate and the *P. multocida* strain from bovines from Chongqing, China, were located on the same small evolutionary clade, with 99.9% identity between the two strains (Figure 5 and Supplementary Figure S6). These results indicated that the bacterial isolate belonged to *P. multocida* and had a closer genetic relationship with the bovine strain.

### 3.7. Species and Capsular Serotype of the Isolated Bacteria

**Figure 6:**
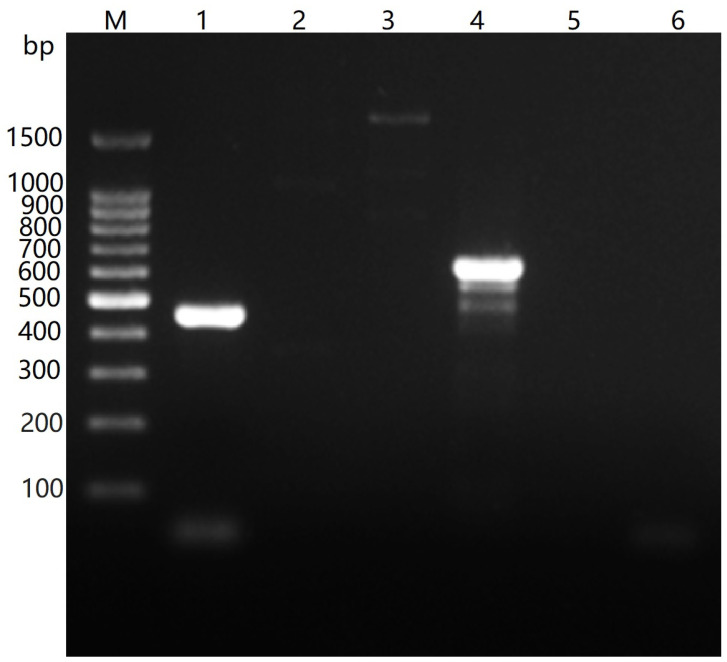
Identification species and capsular serotypes of the isolated bacteria by PCR. M: DNA Ladder; 1: Species specific gene; 2~6: Capsular serotype A, B, D, E and F.

In order to further identify the species and capsular serotype of the bacterial isolate, PCR was employed for amplification of species and serotype-specific nucleotide fragments of *P. multocida*, using the genomic DNA of the isolate as templates, and the specific primers of bacterial species and capsular serotype, respectively. A specific nucleotide fragment of 460 bp matching the expected size could be amplified when using *P. multocida*-specific primers for PCR. The expected products of 657 bp size were obtained by PCR with specific primers of the capsular serotype D of *P. multocida*, but no target gene fragments were detected with specific primers of the other four (A, B, E and F) serotypes (Figure 6). The results indicated that the isolated strain belonged to the *P. multocida* capsular serotype D.

### 3.8. Pathogenicity of the Isolated Bacteria

**Figure 7:**
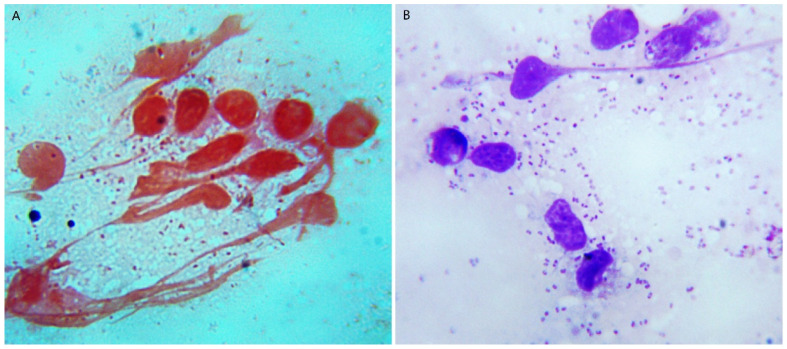
Microscopic examination of bacteria in lung smears of infected mice. (**A**) Gram stain; (**B**) Wright stain.

Animal experiments were carried out to detect the pathogenicity of the bacterial isolate HBShz_2021. Four infected mice were lethargic and stopped eating 6 h after infection, and all died 24–27 h post infection, with a mortality rate of 100%. Necropsy showed obvious congestion and bleeding in the lungs and liver. Microscopically, numerous short Gram-negative bacilli could be seen in the lung, liver, and kidney tissues (Figure 7). The bacteria were isolated from the above tissues with the same cultural and morphological characteristics as the original isolated strains. All the negative control mice were normal and no bacteria were detected in their tissues.

## 4. Discussion

Respiratory diseases in nursery, growing, and finishing pig herds are one of the main causes of reductions in the economic benefits of pig farms. A variety of pathogens are associated with respiratory diseases, such as viruses, bacteria, and parasites, among which viruses and bacteria are the main causes. Viruses like PRRSV, PCV2, PRV, PCV3, swine influenza viruses, and bacteria such as *M. hyopneumoniae*, *A. pleuropneumoniae*, *P. multocida*, *Glaeserella parasuis*, and *S. suis* are often responsible for the development of clinical respiratory diseases in pig farms [13,30,31]. However, the types of pathogens causing respiratory diseases and the mixed infection of diverse pathogens often change with changes in herds, time, and place, which causes great difficulties for the diagnosis, control, and treatment of diseases [32]. In most pig farms, the etiology of respiratory diseases is rarely identified, resulting in poor clinical treatment. Therefore, systematic laboratory diagnosis of respiratory diseases is very necessary. In this study, we first excluded swine influenza and enzootic pneumonia based on epidemiological investigation, clinical manifestations, and anatomical lesions. Even so, based on the prevalence of *M. pneumoniae* infection and its important role in PRDC, we first demonstrated the absence of *M. pneumoniae* infection in the lungs by PCR before bacterial isolation and identification. A series of laboratory tests were then carried out, which proved that the respiratory disease of a growing pig herd was caused by the mixed infection of PCV2a genotype and *P. multocida* capsule serotype D. This means that it would be beneficial for pig farms to take targeted control measures to reduce economic losses.

Subsequently, we examined PRRSV, PCV2, PCV3, and PRV, which are frequent causes of respiratory diseases. Only the ORF2 nucleotide fragment and the whole genome sequence of PCV2 were amplified from the samples to be tested, while no nucleic acids of other viruses were detected. Meanwhile, PCV2 was isolated from the spleen and lymph nodes of the diseased pigs. Compared with the genomic sequence of the PCV2 reference strain, the mutated region of the detected PCV2 strain was mainly in ORF2, which was consistent with many reports [2,11,33].

The high infection rate of PCV2 in the pig population, persistent epidemics, and the mutability of the virus genome make epidemic PCV2 strains complex in pig farms. There are not only new genotypes, gene mutations, and recombinant PCV2 strains, but also the co-infection of different genotypes in the same pig population [8,11,34]. Currently, PCV2 genotypes common in China and other parts of the world mainly include PCV2a, PCV2b, PCV2d, and PCV2e. The PCV2d genotype is dominant, followed by PCV2b, and PCV2a and PCV2e have been detected in a few pig farms [30,31,35,36]. The PCV2b and PCV2d strains were prevalent in some pig farms in Hebei Province, China, from 2004 to 2014. However, two other genotype strains, PCV2a and PCV2e, were detected in addition to PCV2b and PCV2d genotypes in 2018, indicating the genotype diversity of PCV2 strains epidemic in this region [23,35]. In this study, we demonstrated that the prevalent PCV2 strain in the infected pig farm was the PCV2a genotype. This is consistent with the epidemic situation in the region in recent years. In addition, these results indicate that although PCV2d is the dominant genotype in this region, other genotypes, such as PCV2a, are still present, suggesting that the circulating PCV2 strains are still complex in pig farms and should be of concern to regulators and practitioners.

Porcine respiratory diseases are usually caused by the coinfection of two or more different pathogens. This coinfection can be caused by either different species or types of virus, bacteria, or virus with bacteria [12,13,17]. PCV2 is one of the most important primary pathogens causing PRDC, which mainly invades lymphoid tissue, resulting in lymphocyte depletion, immunosuppression or immune dysfunction in pigs, and eventually leads to systemic multiple inflammations, such as interstitial pneumonia, myocarditis, hepatitis, nephritis, and enteritis [3,4,12]. The immunosuppression induced by PCV2 mainly manifests as a decrease in phagocytosis and the antigen-presentation function of alveolar macrophages and dendritic cells; the disturbance of the cytokine-expression profile, such as the significant increase in IL-10 expression; and the decrease in type I or II interferon and IL-12 expression, which create favorable conditions for the secondary infection of other pathogenic microorganisms [37,38,39,40,41]. Therefore, in PCV2-positive pigs, secondary or mixed bacterial infections often occur and cause corresponding clinical symptoms. *P. multocida* is a common pathogen of porcine respiratory diseases, and it, as a secondary pathogen, frequently coordinates with the primary pathogen PCV2 to cause respiratory diseases [13,22,42,43]. Various serotypes and dominant serotypes of *P. multocida* may be prevalent in different regions. Serotypes A, D, and B are common in most regions, especially A and D [22,44,45]. In this study, *P. multocida* serotype D and PCV2a genotype strains were simultaneously isolated from pigs suffering from respiratory disease in this area for the first time. The bacterial strain was most closely related to a bovine strain with an 99.9% identity between them, suggesting that the isolated strain might come from cattle. However, it is not clear how PCV2a or *P. multocida* serotype D strains were introduced into the farm, that is, how pigs were exposed to these two pathogenic microorganisms. These pathogens may come from the pig farm, or from outside the pig farm due to breeders, vehicles, or the introduction of breeding stock. In addition, breeders or vehicles that go outside the farm are likely to come into contact with other animals such as cattle that may carry *Pasteurella multocida*. Therefore, it is difficult to know how these pigs were exposed to these two pathogens and developed PRDC. This result suggests that pig farms should strengthen biosecurity management in order to effectively prevent the introduction and cross-species transmission of pathogens.

It should be pointed out that although the isolated strain was inferred to have a close genetic relationship with a bovine *P. multocida* strain based on the 16S rDNA sequence in this study, the high similarity of 16 s rDNA sequence may not accurately reflect the relations between different isolates. Multilocus sequence typing (MLST) and core genome multilocus sequence typing (cgMLST) are by far the most effective approaches for identifying bacterial isolates or classifying bacterial strains [46,47]. Therefore, the MLST or cgMLST approaches is still needed to further identify the bacterial isolates.

In addition, considering that the purpose of this study was to identify the pathogenic microorganism responsible for PRDC, no antibiotic sensitivity test was performed when the experiment was designed at that time. However, an antibiotic sensitivity test is of great significance for clinical control of secondary bacterial infections. Therefore, in the next step, we will detect the antibiotic sensitivity of *P. multocida* isolates or analyze their drug resistance genes, so as to provide a valuable reference for clinical drug treatment.

Based on the preliminary clinical diagnosis and laboratory test results, it was demonstrated that the co-infection of genotype PCV2a and *P. multocida* serotype D was responsible for the respiratory disease in this pig herd, in which PCV2a is likely the primary pathogen and *P. multocida* may be the secondary pathogen. The results of the study could provide significant help for the effective control and treatment of this disease in pig farms.

## 5. Conclusions

PCV2a and *P. multocida* capsular serotype D strains were detected and isolated from the lung, spleen, and lymph-node tissue of growing pigs with respiratory disease in a pig farm, but PRRSV, PCV3, PRV, and *M. hyopneumoniae* were not detected. The study indicates that their co-infection is likely responsible for this respiratory disease. It also suggests that the PCV2a genotype strain is still present and causing harm in some pig farms in the region. The study could provide important etiological information for effective control and treatment of the disease in pig farms.

## Figures and Tables

**Table 1:** Primers for pathogen gene amplification.

Virus/Gene	Primer	Sequence (5′→3′)	Ta (°C)	Amplicon Size (bp)	Reference
PCV2/cap	PCV2_cap_U PCV2_cap_L	ACGGATATTGTAGTCCTGGT CAAGGCTACCACAGTCAGAA	57	472	This study
PCV2/genome	PCV2_U	GCTGGCTGAACTTTTGAAAGT	48	1767	Li et al. (2016) [23]
	PCV2_L	AAATTTCTGACAAACGTTACA			
PCV3/cap	PCV3_cap_U PCV3_cap_L	ACATGCGAGGGCGTTTAC CACTTCTGGCGGGAACTA	56	307	This study
PRRSV/orf7	orf7_U orf7_L	ATGGCCAGCCAGTCAATCAG ATGCTGAGGGTGACGTTGTG	56	327	This study
PRV/gE	gE_U gE_L	ACGGCGACCTCGACGGCGAC CCCGAGGCGTCGTGCAGCGT	67	418	This study
*M.hyopneumoniae* /Intergenic space	M.hyopnU	CGGTTTTATAAGAATTAGTTGCTCC	58	421	Lung et al. (2017) [24]
	M.hyopnL	TTGGCAAGCCGCCGTCATT			

**Table 2:** The information of PCV2 reference strains used in this study.

Strain	GenBank ID	Geographic Origin	Submitted Time	Genome Size (bp)	Genotype
LG	HM038034	Heilongjiang, China	2010	1768	PCV2a
JXPX-1-1	KJ437192	Hunan, China	2014	1768	PCV2a
BJ0602	EF524540	Beijing, China	2007	1768	PCV2a
SH0901	KF850458	Shanghai, China	2013	1768	PCV2a
n32na	DQ629113	USA	2006	1768	PCV2a
DTC	DQ104423	Jiangsu, China	2005	1768	PCV2a
BF	AF381175	Beijing, China	2001	1768	PCV2a
HSH1	DQ910866	Hebei, China	2006	1767	PCV2b
K43C	DQ629118	USA	2006	1767	PCV2b
Bd3-2007	KM624031	Hebei, China	2014	1767	PCV2b
XZBK	KF926650	Henan, China	2013	1767	PCV2b
HB0802	FJ608549	Hebei, China	2008	1767	PCV2b
Fh19	AY322003	France	2003	1767	PCV2b
TY4	HQ202967	Taiwan, China	2010	1767	PCV2b
DK1980PMWSfree	EU148503	Denmark	2007	1767	PCV2c
DK1990PMWSfree	EU148505	Denmark	2007	1767	PCV2c
TJ06	EF524539	Tianjin, China	2007	1767	PCV2d
TJ	AY181946	Tianjin, China	2002	1767	PCV2d
HX	KC860786	Heilongjiang, China	2013	1767	PCV2d
JSTZ	JQ413808	Jiangsu, China	2012	1767	PCV2d
HbTs2011	KM624037	Hebei, China	2014	1767	PCV2d
BDMC1/2016	KY613030	Hebei, China	2017	1767	PCV2d
BDAG2015	KY569382	Hebei, China	2017	1767	PCV2d

**Table 3:** The information of the reference bacterial strains.

Strain	GenBank ID	Host	Organism	Country
9	FJ463827	chicken	*P.* *multocida*	South Korea
-	E05329	-	*P.* *multocida*	Japan
4074	FJ405341	pig	*P.* *multocida*	South Korea
F	CP013291	bovine	*P.* *multocida*	China
4075	FJ405342	goat	*P.* *multocida*	South Korea
Tibet-Pm1	CP072655	yak	*P.* *multocida*	China
PM-1	CP066223	yak	*P.* *multocida*	China
ATCC 43325	NR_118749	-	*P.* *multocida*	USA
LSBS1	MN080875	Moschus berezovskii	*P.* *multocida*	China
BD1643	LC619668	Oryctolagus cuniculus	*P.* *multocida*	Japan
PH40	U57070	bovine	*P.* *haemolytica*	USA
CCUG 28148	AF224287	-	*P.* *haemolytica*	USA
PH704	U57072	bovine	*P.* *haemolytica*	USA
PH2, PH8	U57066	sheep, bovine	*P.* *haemolytica*	USA
xws8	KT588712	pig	*Glaeserella* *parasuis*	China
h1-6	KT588715	pig	*Glaeserella* *parasuis*	China
5651	JSVY01000046	pig	*A. pleuropneumoniae*	Brazil
597	JSVX01000052	pig	*A. pleuropneumoniae*	Brazil

**Table 4:** Primers for the detection of capsular serotype of *P.m* [28].

Serotype	Gene	Primer	Sequence (5′→3′)	Amplicon Size (bp)
All	KMT1	*P. m*^a^_U *P. m*_L	TGCCAAAATCGCAGTCAG TTGCCATCATTGTCAGTG	460
A	*hyaD-hyaC*	CAPA_U CAPA_L	TGCCAAAATCGCAGTCAG TTGCCATCATTGTCAGTG	1044
B	*bcbD*	CAPB_U CAPB_L	CATTTATCCAAGCTCCACC GCCCGAGAGTTTCAATCC	760
D	*dcbF*	CAPD_U CAPD_L	TTACAAAAGAAAGACTAGGAGCCC CATCTACCCACTCAACCATATCAG	657
E	*ecbJ*	CAPE_U CAPE_L	TCCGCAGAAAATTATTGACTC GCTTGCTGCTTGATTTTGTC	511
F	*fcbD*	CAPF_U CAPF_L	AATCGGAGAACGCAGAAATCAG TTCCGCCGTCAATTACTCTG	851

^a^*P. m* stands for *P. multocida*.

**Table 5:** Biochemical characteristics of the isolated bacteria.

Item	Reaction	Criteria of *P.* *multocida* ^a^
Glucose	− ^b^	+
Lactose	−	(+) ^d^
Maltose	−	(−)
Sucrose	+ ^c^	+
Mannitol	+	(+)
Dulcitol	−	/ ^e^
Xylose	+	/
Sorbitol	+	/
Adonitol	−	/
Raffinose	−	/
Urease	−	−
Catalase	+	+
Indole	+	+
Hydrogen sulfide	+	+
Indole	+	+
Methyl red (MR)	−	/
Voges–Proskaurer (VP)	−	/
Citrate	−	/
Phenylalanine decarboxylase	−	/
Ornithine decarboxylase	−	(+)
Lysine decarboxylase	−	/

^a^ Result indicated by Bergey’s Manual of Systematic Bacteriology (9th edition). ^b^ Negative reaction; ^c^ Positive reaction; ^d^ Majority; ^e^ No data.

## Data Availability

The data of this study are available from the corresponding author upon reasonable request.

## Supplementary Materials

The following supporting information can be downloaded at: https://www.mdpi.com/article/10.3390/vetsci9100528/s1, Figure S1: Identity analysis of complete genome sequences between PCV2 HBSZ-2021 strain and the reference strains; Figure S2: Identity analysis of ORF1 between HBSZ-2021 strain and PCV2 reference strains of different genotypes; Figure S3: Homology analysis of ORF2 between HBSZ-2021 and PCV2 reference strains of different genotypes; Figure S4: Phylogenetic trees of PCV2 HBSZ-2021 strain and the reference strains based on the genome sequences; Figure S5: Culture characteristics of the bacterial isolate; Figure S6: Homology analysis of the bacterial isolate and the reference strains based on 16S rDNA sequence.

## References

[1] Rosario K., Breitbart M., Harrach B., Segales J., Delwart E., Biagini P., Varsani A. (2017). Revisiting the taxonomy of the family Circoviridae: Establishment of the genus Cyclovirus and removal of the genus Gyrovirus. Arch. Virol..

[2] Kekarainen T., Segales J. (2015). Porcine circovirus 2 immunology and viral evolution. Porcine Health Manag..

[3] Chae C. (2005). A review of porcine circovirus 2-associated syndromes and diseases. Vet. J..

[4] Opriessnig T., Langohr I. (2013). Current state of knowledge on porcine circovirus type 2-associated lesions. Vet. Pathol..

[5] Opriessnig T., Halbur P.G. (2012). Concurrent infections are important for expression of porcine circovirus associated disease. Virus Res..

[6] Ouyang T., Zhang X., Liu X., Ren L. (2019). Co-Infection of Swine with Porcine Circovirus Type 2 and Other Swine Viruses. Viruses.

[7] Firth C., Charleston M.A., Duffy S., Shapiro B., Holmes E.C. (2009). Insights into the evolutionary history of an emerging livestock pathogen: Porcine circovirus 2. J. Virol..

[8] Franzo G., Segales J. (2018). Porcine circovirus 2 (PCV-2) genotype update and proposal of a new genotyping methodology. PLoS ONE.

[9] Li N., Liu J., Qi J., Hao F., Xu L., Guo K. (2021). Genetic Diversity and Prevalence of Porcine Circovirus Type 2 in China During 2000–2019. Front. Vet. Sci..

[10] Bao F., Mi S., Luo Q., Guo H., Tu C., Zhu G., Gong W. (2018). Retrospective study of porcine circovirus type 2 infection reveals a novel genotype PCV2f. Transbound. Emerg. Dis..

[11] Kang L., Wahaab A., Shi K., Mustafa B.E., Zhang Y., Zhang J., Li Z., Qiu Y., Li B., Liu K. (2022). Molecular Epidemic Characteristics and Genetic Evolution of Porcine Circovirus Type 2 (PCV2) in Swine Herds of Shanghai, China. Viruses.

[12] Chae C. (2016). Porcine respiratory disease complex: Interaction of vaccination and porcine circovirus type 2, porcine reproductive and respiratory syndrome virus, and Mycoplasma hyopneumoniae. Vet. J..

[13] Saade G., Deblanc C., Bougon J., Marois-Crehan C., Fablet C., Auray G., Belloc C., Leblanc-Maridor M., Gagnon C.A., Zhu J. (2020). Coinfections and their molecular consequences in the porcine respiratory tract. Vet. Res..

[14] Zhao D., Yang B., Yuan X., Shen C., Zhang D., Shi X., Zhang T., Cui H., Yang J., Chen X. (2021). Advanced Research in Porcine Reproductive and Respiratory Syndrome Virus Co-infection With Other Pathogens in Swine. Front. Vet. Sci..

[15] Obradovic M.R., Segura M., Segales J., Gottschalk M. (2021). Review of the speculative role of co-infections in Streptococcus suis-associated diseases in pigs. Vet. Res..

[16] Choi Y.K., Goyal S.M., Joo H.S. (2003). Retrospective analysis of etiologic agents associated with respiratory diseases in pigs. Can. Vet. J..

[17] Kim K.S., Jung J.Y., Kim J.H., Kang S.C., Hwang E.K., Park B.K., Kim D.Y., Kim J.H. (2011). Epidemiological characteristics of pulmonary pneumocystosis and concurrent infections in pigs in Jeju Island, Korea. J. Vet. Sci..

[18] Vangroenweghe F., Thas O. (2021). Seasonal Variation in Prevalence of Mycoplasma hyopneumoniae and Other Respiratory Pathogens in Peri-Weaned, Post-Weaned, and Fattening Pigs with Clinical Signs of Respiratory Diseases in Belgian and Dutch Pig Herds, Using a Tracheobronchial Swab Sampling Technique, and Their Associations with Local Weather Conditions. Pathogens.

[19] Turni C., Meers J., Parke K., Singh R., Yee S., Templeton J., Mone N.K., Blackall P.J., Barnes T.S. (2021). Pathogens associated with pleuritic pig lungs at an abattoir in Queensland Australia. Aust. Vet. J..

[20] Valeris-Chacin R., Sponheim A., Fano E., Isaacson R., Singer R.S., Nerem J., Leite F.L., Pieters M. (2021). Relationships among Fecal, Air, Oral, and Tracheal Microbial Communities in Pigs in a Respiratory Infection Disease Model. Microorganisms.

[21] Smith E., Miller E., Aguayo J.M., Figueroa C.F., Nezworski J., Studniski M., Wileman B., Johnson T. (2021). Genomic diversity and molecular epidemiology of Pasteurella multocida. PLoS ONE.

[22] Liu H., Zhao Z., Xi X., Xue Q., Long T., Xue Y. (2017). Occurrence of Pasteurella multocida among pigs with respiratory disease in China between 2011 and 2015. Ir. Vet. J..

[23] Li L., Yuan W., Guo H., Ma Z., Song Q., Wang X., Li H. (2016). Prevalence and genetic variation of porcine circovirus type 2 in Hebei, China from 2004 to 2014. Gene.

[24] Lung O., Ohene-Adjei S., Buchanan C., Joseph T., King R., Erickson A., Detmer S., Ambagala A. (2017). Multiplex PCR and Microarray for Detection of Swine Respiratory Pathogens. Transbound. Emerg. Dis..

[25] Truong C., Mahe D., Blanchard P., Le Dimna M., Madec F., Jestin A., Albina E. (2001). Identification of an immunorelevant ORF2 epitope from porcine circovirus type 2 as a serological marker for experimental and natural infection. Arch. Virol..

[26] Wang Y., Guo H., Bai Y., Li T., Xu R., Sun T., Lu J., Song Q. (2020). Isolation and characteristics of multi-drug resistant Streptococcus porcinus from the vaginal secretions of sow with endometritis. BMC Vet. Res..

[27] Meena B., Rajan L.A., Vinithkumar N.V., Kirubagaran R. (2013). Novel marine actinobacteria from emerald Andaman & Nicobar Islands: A prospective source for industrial and pharmaceutical byproducts. BMC Microbiol..

[28] Townsend K.M., Boyce J.D., Chung J.Y., Frost A.J., Adler B. (2001). Genetic organization of Pasteurella multocida cap Loci and development of a multiplex capsular PCR typing system. J. Clin. Microbiol..

[29] Mostaan S., Ghasemzadeh A., Asadi Karam M.R., Ehsani P., Sardari S., Shokrgozar M.A., Abolhassani M., Nikbakht Brujeni G. (2021). Pasteurella multocida PlpE Protein Polytope as a Potential Subunit Vaccine Candidate. Vector Borne Zoonotic Dis..

[30] Yue W., Liu Y., Meng Y., Ma H., He J. (2021). Prevalence of porcine respiratory pathogens in slaughterhouses in Shanxi Province, China. Vet. Med. Sci..

[31] Xu T., Zhang Y.H., Tian R.B., Hou C.Y., Li X.S., Zheng L.L., Wang L.Q., Chen H.Y. (2021). Prevalence and genetic analysis of porcine circovirus type 2 (PCV2) and type 3 (PCV3) between 2018 and 2020 in central China. Infect. Genet. Evol..

[32] Weissenbacher-Lang C., Kureljusic B., Nedorost N., Matula B., Schiessl W., Stixenberger D., Weissenbock H. (2016). Retrospective Analysis of Bacterial and Viral Co-Infections in Pneumocystis spp. Positive Lung Samples of Austrian Pigs with Pneumonia. PLoS ONE.

[33] Dinh P.X., Nguyen M.N., Nguyen H.T., Tran V.H., Tran Q.D., Dang K.H., Vo D.T., Le H.T., Nguyen N.T.T., Nguyen T.T. (2021). Porcine circovirus genotypes and their copathogens in pigs with respiratory disease in southern provinces of Vietnam. Arch. Virol..

[34] Jang G., Yoo H., Kim Y., Yang K., Lee C. (2021). Genetic and phylogenetic analysis of porcine circovirus type 2 on Jeju Island, South Korea, 2019-2020: Evidence of a novel intergenotypic recombinant. Arch. Virol..

[35] Han L., Yuan G.F., Chen S.J., Dai F., Hou L.S., Fan J.H., Zuo Y.Z. (2021). Porcine circovirus type 2 (PCV2) infection in Hebei Province from 2016 to 2019: A retrospective study. Arch. Virol..

[36] Liu Y., Gong Q.L., Nie L.B., Wang Q., Ge G.Y., Li D.L., Ma B.Y., Sheng C.Y., Su N., Zong Y. (2020). Prevalence of porcine circovirus 2 throughout China in 2015-2019: A systematic review and meta-analysis. Microb. Pathog..

[37] Vincent I.E., Carrasco C.P., Guzylack-Piriou L., Herrmann B., McNeilly F., Allan G.M., Summerfield A., McCullough K.C. (2005). Subset-dependent modulation of dendritic cell activity by circovirus type 2. Immunology.

[38] Doster A.R., Subramaniam S., Yhee J.Y., Kwon B.J., Yu C.H., Kwon S.Y., Osorio F.A., Sur J.H. (2010). Distribution and characterization of IL-10-secreting cells in lymphoid tissues of PCV2-infected pigs. J. Vet. Sci..

[39] Kang S.J., Park I.B., Chun T. (2021). Open reading frame 5 protein of porcine circovirus type 2 induces RNF128 (GRAIL) which inhibits mRNA transcription of interferon-beta in porcine epithelial cells. Res. Vet. Sci..

[40] Wang Z., Chen J., Zhang Q.G., Huang K., Ma D., Du Q., Tong D., Huang Y. (2022). Porcine circovirus type 2 infection inhibits the activation of type I interferon signaling via capsid protein and host gC1qR. Vet. Microbiol..

[41] Du Q., Wu X., Wang T., Yang X., Wang Z., Niu Y., Zhao X., Liu S.L., Tong D., Huang Y. (2018). Porcine Circovirus Type 2 Suppresses IL-12p40 Induction via Capsid/gC1qR-Mediated MicroRNAs and Signalings. J. Immunol..

[42] Cheong Y., Oh C., Lee K., Cho K.H. (2017). Survey of porcine respiratory disease complex-associated pathogens among commercial pig farms in Korea via oral fluid method. J. Vet. Sci..

[43] Hansen M.S., Pors S.E., Jensen H.E., Bille-Hansen V., Bisgaard M., Flachs E.M., Nielsen O.L. (2010). An investigation of the pathology and pathogens associated with porcine respiratory disease complex in Denmark. J. Comp. Pathol..

[44] Vu-Khac H., Trinh T.T.H., Nguyen T.T.G., Nguyen X.T., Nguyen T.T. (2020). Prevalence of virulence factor, antibiotic resistance, and serotype genes of Pasteurella multocida strains isolated from pigs in Vietnam. Vet. World.

[45] Devi L.B., Bora D.P., Das S.K., Sharma R.K., Mukherjee S., Hazarika R.A. (2018). Virulence gene profiling of porcine Pasteurella multocida isolates of Assam. Vet. World.

[46] Alikhan N.F., Zhou Z., Sergeant M.J., Achtman M. (2018). A genomic overview of the population structure of Salmonella. PLoS Genet..

[47] Hennart M., Guglielmini J., Bridel S., Maiden M.C.J., Jolley K.A., Criscuolo A., Brisse S. (2022). A Dual Barcoding Approach to Bacterial Strain Nomenclature: Genomic Taxonomy of Klebsiella pneumoniae Strains. Mol. Biol. Evol..

