# Supplementary material for: Detection of Porcine Circovirus Type 2a and Pasteurella multocida Capsular Serotype D in Growing Pigs Suffering from Respiratory Disease
Source: Vet Sci. 2022 Sep 27;9(10):528. doi: 10.3390/vetsci9100528 (PMC9607208; doi:10.3390/vetsci9100528)
Supplement: Supplementary file 1 [file vetsci-09-00528-s001.zip › vetsci-1874522-Supplementary files R 2022.9.26.pdf]

Supplementary files

Percent Identity (above the diagonal); Divergence (below the diagonal)

	1	2	3	4	5	6	7	8	9	10	11	12	13	14	15	16	17	18	19	20	21	22	23	24		
1		97.3	97.2	97.1	97.5	96.5	97.4	96.9	95.8	95.2	94.8	95.3	95.2	95.5	95.2	93.6	93.5	94.9	94.5	94.6	94.3	94.2	94.5	94.4	1	MZ161162_HBSZ-2021
2	2.7		98.6	98.8	99.7	97.1	99.0	97.0	96.3	95.9	95.4	95.9	95.9	96.0	95.8	94.5	94.2	95.9	95.2	95.2	95.0	94.9	95.0	94.9	2	AF381175_BF
3	2.8	1.4		98.9	98.7	96.9	98.8	96.7	96.2	96.0	95.6	95.9	95.9	95.8	96.0	94.4	94.2	95.6	95.0	95.0	94.8	94.7	94.8	94.7	3	DQ104423_DTC
4	2.9	1.2	1.1		98.8	96.7	98.9	96.7	95.9	95.6	95.2	95.6	95.7	95.6	95.6	94.2	94.0	95.4	94.9	95.0	94.7	94.6	94.7	94.7	4	DQ62911_n32na
5	2.6	0.3	1.3	1.2		97.1	99.2	97.1	96.3	95.9	95.4	95.9	95.9	96.0	95.8	94.5	94.2	95.9	95.2	95.2	95.0	94.9	95.0	94.9	5	EF524540_BJ0602
6	3.5	3.0	3.2	3.4	3.0		96.8	96.5	96.1	95.6	95.2	95.6	96.5	95.6	95.8	94.2	93.9	95.8	95.3	95.5	95.3	95.2	95.3	95.2	6	HM038034_LG
7	2.6	1.0	1.2	1.1	0.8	3.2		96.7	96.2	95.7	95.2	95.6	95.6	95.8	95.6	94.2	93.9	95.7	95.4	95.2	95.0	94.9	95.0	94.9	7	KF850458_SH0901
8	3.1	3.1	3.4	3.4	2.9	3.5	3.4		96.2	95.5	95.2	95.8	95.8	96.0	95.6	94.1	94.2	95.5	95.0	95.0	94.9	94.7	94.9	94.8	8	KJ437192_JXPX-1-1
9	4.3	3.8	3.9	4.3	3.8	4.0	4.0	4.0		99.0	98.7	98.3	98.6	98.5	99.1	95.3	95.3	96.9	96.1	96.2	95.9	95.8	95.8	95.8	9	AY322003_Fh19
10	4.9	4.3	4.1	4.5	4.3	4.6	4.4	4.6	1.0		99.1	98.2	98.6	98.3	99.5	94.7	94.7	96.5	95.6	95.9	95.6	95.6	95.5	95.5	10	DQ629118_k43C
11	5.4	4.7	4.6	4.9	4.7	4.9	4.9	4.9	1.3	0.9		98.0	98.3	98.0	99.2	94.6	94.6	96.1	95.2	95.5	95.3	95.2	95.2	95.1	11	DQ910866_HSH1
12	4.9	4.3	4.2	4.5	4.3	4.5	4.5	4.4	1.7	1.8	2.0		98.5	99.2	98.5	95.0	94.9	96.6	95.9	96.2	96.0	95.9	95.9	95.8	12	FJ608549_HB0802
13	4.9	4.3	4.3	4.4	4.3	3.6	4.6	4.3	1.4	1.4	1.7	1.5		98.4	98.8	95.2	95.1	96.9	96.2	96.4	96.2	96.0	96.0	96.0	13	HQ202967_TY4
14	4.7	4.1	4.3	4.6	4.1	4.5	4.4	4.1	1.5	1.7	2.1	0.9	1.6		98.4	95.0	95.0	96.5	95.9	96.3	96.2	96.0	96.0	95.9	14	KF926650_XZBK
15	5.0	4.3	4.1	4.5	4.3	4.4	4.5	4.5	0.9	0.5	0.8	1.5	1.2	1.7		94.9	94.9	96.7	95.7	96.0	95.8	95.8	95.6	95.6	15	KM624031_Bd3-2007
16	6.7	5.8	5.8	6.0	5.8	6.0	6.1	6.1	4.9	5.5	5.6	5.2	5.0	5.2	5.3		99.0	95.1	94.3	94.3	94.1	94.0	94.1	94.0	16	EU148503_DK1980PMWSfree
17	6.8	6.0	6.1	6.3	6.0	6.3	6.3	6.1	4.9	5.5	5.6	5.3	5.0	5.2	5.3	1.0		95.0	94.3	94.3	94.1	94.0	94.1	94.0	17	EU148505_DK1990PMWSfree
18	5.3	4.3	4.5	4.7	4.3	4.4	4.4	4.7	3.1	3.5	4.0	3.5	3.1	3.5	3.4	5.0	5.2		98.4	98.1	97.9	97.9	97.7	97.7	18	AY181946_TJ
19	5.7	4.9	5.2	5.3	4.9	4.9	4.8	5.2	4.0	4.6	4.9	4.2	3.9	4.2	4.4	5.9	5.9	1.6		99.0	98.8	98.6	98.6	98.5	19	EF524539_TJ06
20	5.6	5.0	5.2	5.2	5.0	4.6	5.0	5.2	4.0	4.3	4.6	3.9	3.7	3.8	4.1	5.9	5.9	1.9	1.0		99.7	99.5	99.5	99.4	20	JQ413808_JSTZ
21	5.9	5.2	5.4	5.5	5.2	4.9	5.2	5.3	4.2	4.5	4.9	4.1	4.0	3.9	4.4	6.1	6.1	2.1	1.3	0.3		99.3	99.3	99.2	21	KC860786_HX
22	6.0	5.3	5.5	5.6	5.3	5.0	5.3	5.5	4.3	4.5	5.0	4.3	4.1	4.1	4.4	6.3	6.3	2.1	1.4	0.5	0.7		99.2	99.1	22	KM624037_HbTs2011
23	5.8	5.2	5.4	5.5	5.2	4.9	5.2	5.3	4.3	4.6	5.0	4.3	4.1	4.1	4.5	6.2	6.2	2.3	1.4	0.5	0.7	0.9		99.8	23	KY569382_BDAG2015
24	5.8	5.3	5.5	5.5	5.3	4.9	5.3	5.4	4.4	4.7	5.0	4.3	4.1	4.2	4.6	6.3	6.3	2.4	1.5	0.6	0.8	0.9	0.2		24	KY613030_BDMC1-2016
	1	2	3	4	5	6	7	8	9	10	11	12	13	14	15	16	17	18	19	20	21	22	23	24		

Figure S1. Identity analysis of complete genome sequences between PCV2 HBSZ-2021 strain and the reference strains. The data above and below the line of black cells refer to the percent identity and difference between the corresponding two sequences, respectively. The information of PCV2 reference strains is shown in Table 2.

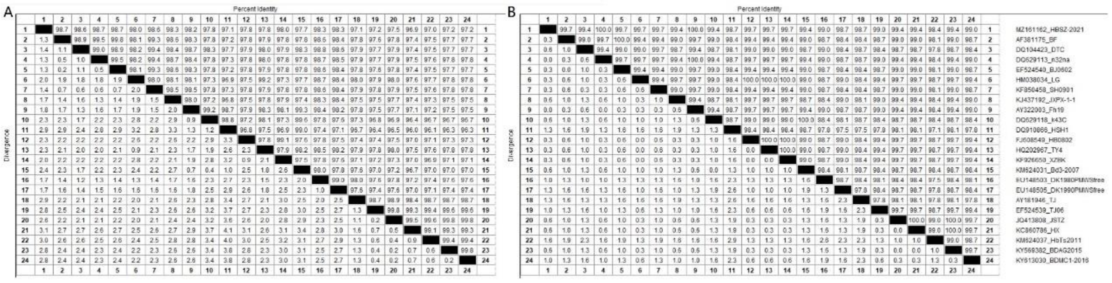

Figure S2. Identity analysis of ORF1 between HBSZ-2021 strain and PCV2 reference strains of different genotypes. A. nucleotide sequence; B. amino acid sequence. The data above and below the line of black cells refer to the percent identity and difference between the corresponding two sequences, respectively. The information of PCV2 reference strains is shown in Table 2.

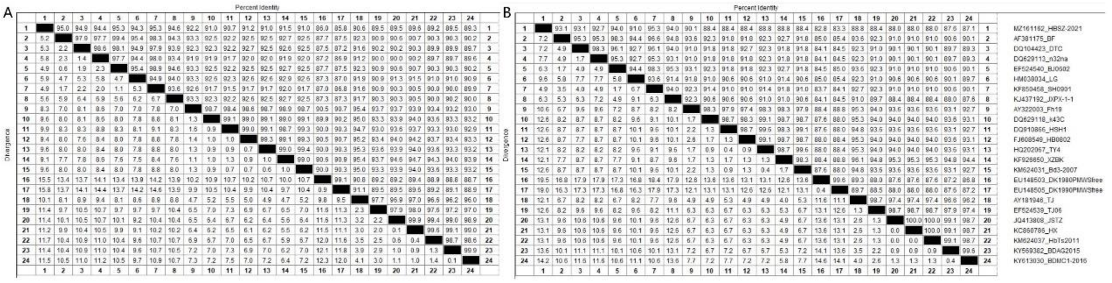

Figure S3. Homology analysis of ORF2 between HBSZ-2021 and PCV2 reference strains of different genotypes. A. nucleotide sequence; B. amino acid sequence. The data above and below the line of black cells refer to the percent identity and difference between the corresponding two sequences, respectively. The information of PCV2 reference strains is shown in Table 2.

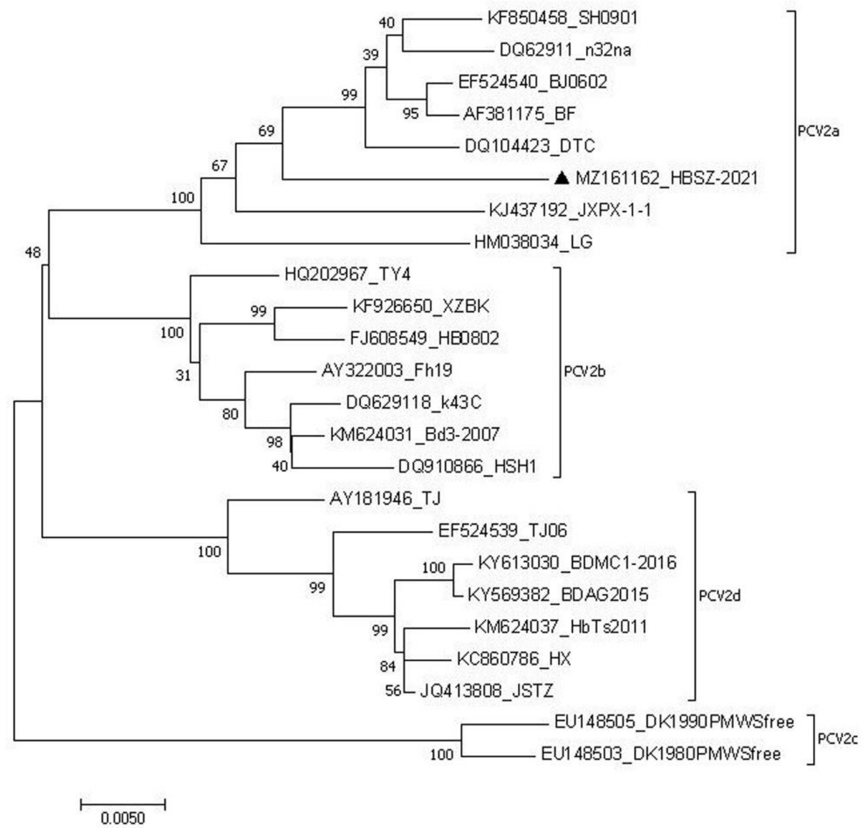

Figure S4. Phylogenetic trees of PCV2 HBSZ-2021 strain and the reference strains based on the genome sequences. The information of PCV2 reference strains is shown in Table 2. ▲The detected strain HBSZ-2021.

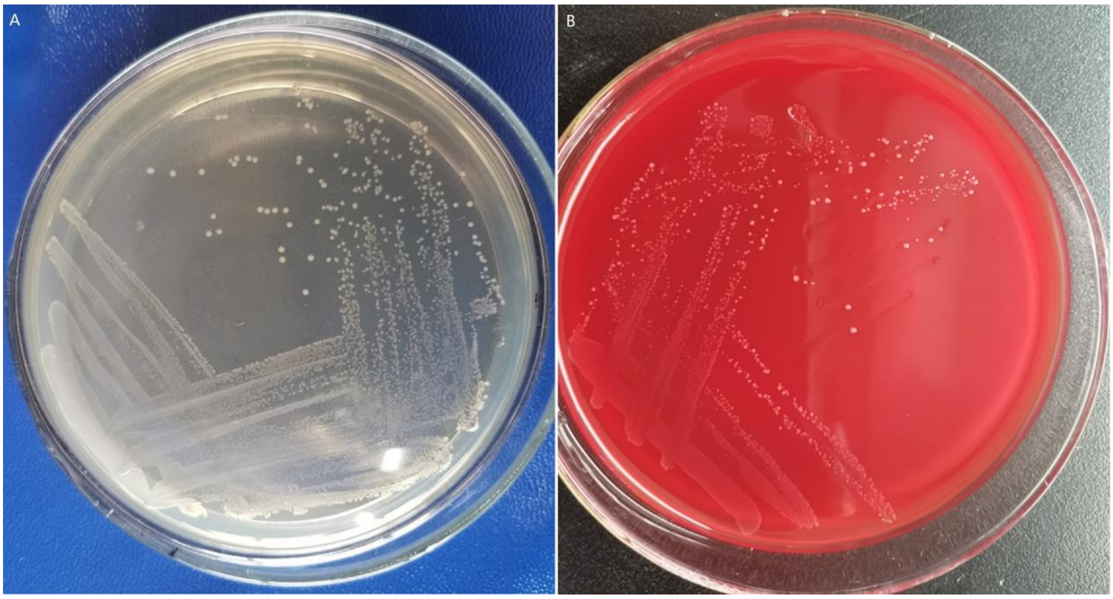

Figure S5. Culture characteristics of the bacterial isolate. A. 5% FBS nutrient agar; B. 5% rabbit blood nutrient agar.

Percent Identity

Divergence

	1	2	3	4	5	6	7	8	9	10	11	12	13	14	15		
1		99.9	99.7	99.7	99.7	99.7	99.4	95.3	99.4	97.6	97.5	93.5	92.5	94.3	94.5	1	OK236057-Pm-Swine-China
2	0.1		99.8	99.8	99.8	99.8	99.5	95.4	99.5	97.7	97.6	93.6	92.6	94.4	94.6	2	CP013291-Pm-Bovine-China
3	0.3	0.2		100.0	99.7	99.7	99.3	95.4	99.5	97.6	97.4	93.6	92.7	94.3	94.7	3	CP072655-Pm-Yak-China
4	0.3	0.2	0.0		99.7	99.7	99.3	95.4	99.5	97.6	97.4	93.6	92.7	94.3	94.7	4	CP066223-Pm-Yak-China
5	0.3	0.2	0.3	0.3		99.7	99.3	95.2	99.3	97.6	97.4	93.5	92.5	94.3	94.6	5	FJ405342-Pm-Native
6	0.3	0.2	0.3	0.3	0.3		99.6	95.2	99.3	97.6	97.4	93.4	92.5	94.3	94.5	6	FJ405341-Pm-Swine-SouthKorea
7	0.6	0.5	0.7	0.7	0.7	0.4		94.9	99.1	97.5	97.2	93.2	92.2	93.9	94.1	7	FJ463827-Pm-Chicken-SouthKorea
8	2.8	2.8	2.8	2.8	3.0	3.0	3.3		96.3	94.5	94.9	93.3	92.1	93.4	94.3	8	NR_118749-Pm-USA
9	0.6	0.5	0.5	0.5	0.7	0.7	1.0	3.1		97.1	98.1	93.0	92.4	93.9	94.0	9	MN080875-Pm-Moschusberezovskii-China
10	2.5	2.3	2.5	2.5	2.4	2.4	2.6	5.0	3.0		96.2	91.4	90.8	92.3	92.3	10	E05329-Pm-Japan
11	2.5	2.5	2.6	2.6	2.7	2.6	2.9	3.2	1.9	3.9		94.3	93.4	94.7	95.2	11	LC619668-Pm-Oryctolaguscuniculus-Japan
12	6.8	6.7	6.7	6.7	6.8	6.9	7.2	6.3	7.3	9.2	5.9		96.4	96.6	97.4	12	U57072-Ph-Bovine-USA
13	7.9	7.8	7.6	7.6	7.9	7.9	8.3	6.9	8.0	9.8	6.9	3.7		95.4	95.9	13	U57066-Ph-Sheep-USA
14	5.7	5.6	5.7	5.7	5.7	5.7	6.0	5.3	6.1	7.8	5.2	3.2	4.5		98.1	14	U57070-Ph-Bovine-USA
15	5.7	5.6	5.5	5.5	5.7	5.7	6.1	5.3	6.3	8.1	4.9	2.7	4.3	1.6		15	AF224287-Ph-USA
	1	2	3	4	5	6	7	8	9	10	11	12	13	14	15		

Figure S6. Homology analysis of the bacterial isolate and the reference strains based on 16S rDNA sequence. The data above and below the line of black cells refer to the percent identity and difference between the corresponding two sequences, respectively. The information of the reference strains is shown in Table 3.
